# The association between experiences of religious discrimination, social-emotional and sleep outcomes among youth in Australia

**DOI:** 10.1016/j.ssmph.2021.100883

**Published:** 2021-07-28

**Authors:** Mienah Z. Sharif, Mandy Truong, Oishee Alam, Kevin Dunn, Jacqueline Nelson, Anne Kavanagh, Yin Paradies, Naomi Priest

**Affiliations:** a Center for the Study of Racism, Social Justice and Health, University of California, Los Angeles, Los Angeles, CA, USA; b Centre for Social Research and Methods, Australian National University, Canberra, Australia; c School of Nursing and Midwifery, Faculty of Medicine, Nursing and Health Sciences, Monash University, Clayton, Australia; d Religion and Society Research Centre, Western Sydney University, Sydney, Australia; e School of Social Sciences, Western Sydney University, Sydney, Australia; f School of Communications, Faculty of Arts and Social Sciences, University of Technology Sydney, Australia; g Disability and Health Unit, Centre for Health Equity, Melbourne School of Population and Global Health, University of Melbourne, Melbourne, Australia; h Faculty of Arts and Education, Deakin University, Burwood, Australia; i Murdoch Children's Research Institute, Melbourne, Australia

**Keywords:** Adolescence, Racism, Religion, Equity, Health promotion

## Abstract

**Background:**

Religious-based hate crimes are on the rise worldwide. However, the relationship of religious discrimination on health and well-being, especially earlier on the lifecourse, is largely understudied. This study examines the prevalence of religious discrimination and the relationship it has on social-emotional adjustment and sleep outcomes among a diverse sample of students in Australia.

**Methods:**

Data came from Speak Out Against Racism, a population-representative cross-sectional study of 4664 public school students in grades 5–9 in Australia in 2017. An adaption of the Adolescent Discrimination Distress Index (ADDI), was used to derive four measures of religious discrimination (peer, school, societal and the sum of those as a “total” score). The Strengths and Difficulties Questionnaire measured the total difficulties, conduct, emotional, and prosocial behavior subscales. Measures of sleep outcomes included duration, latency, and disruption.

**Results:**

27 % (95 % CI 22.82, 31.12) of students reported experiences of direct total religious discrimination with higher levels being reported by students identifying as a religious minority. There was strong evidence that experiences of religious discrimination (across all four sources) was related to all measures of socioemotional adjustment and sleep outcomes.

**Discussion:**

Religious discrimination is an understudied form of social disadvantage that has implications for adolescents’ development, health and well-being. Conclusion: More programs, particularly in the school-context, address religious-based discrimination may reduce inequities in health.

## Highlights

• There is a noted gap in population health research examining how religion operates as a structural determinant of health.
• The relationship between discrimination and indicators of health and wellbeing have been understudied among young children.
• Religious discrimination is associated with poor indicators of social-emotional adjustment across all four measures.
• Religious discrimination is associated with adverse sleep outcomes (duration and latency) among young school-aged children.

## 1 Introduction

Reports of religious discrimination are on the rise, around the world, and is a dominant topic in political discourse particularly in settler-colonial contexts including the United States (U.S.) and Australia (Guardian, 2020). In the US, religious-based hate crimes constitute 22 % of hate crimes, disproportionately affecting people of Jewish and Muslim faiths (Ford, 2019). Anti-Muslim racism is a noted social issue both in the United States and in Australia, with increasing numbers of more serious, targeted incidents (Dunn et al., 2016; Mansouri & Vergani, 2018) that has implications for health and health equity. With the rise in religious-based hate crime around the world, particularly related to Islamophobia (Ford, 2019; Daulatzai & Rana, 2018) the problem of religious discrimination targeting religious minorities in the West shows no sign of abating. Therefore, examining the health impacts of religious discrimination is a population health priority.

To date, quantitative research on religion and health has primarily conceptualized religion as a protective, or health promoting, factor and has focused on individual-level factors including religious participation or religious coping (Klocker et al., 2011). This work has documented positive implications of religious participation and/or religiosity. Higher degrees of religious participation and/or religiosity are associated with a wide range of positive mental health outcomes including less depressive symptoms (Mouzon, 2017), lower levels of serious psychological distress (Chatters et al., 2015) and overall lower mortality risk (George et al., 2002).

However, given the global rise in religious-based hate crimes a noticeable gap in the scientific literature is the multiple directions by which religion can influence health. Religion can simultaneously be a protective factor and “the social problem itself”, that is, a social identity that can lead to discrimination, particularly for religious minorities, and/or can be manipulated (by some) to justify the mistreatment or exclusion of others, such as through patriarchal beliefs that promote subjugation of women or dismissal of science (Popescu et al., 2009). The ways in which religion can be a source of potential risk to some groups’ wellbeing, but also a potential benefit, is a research priority. Religious discrimination represents how religion may become problematic for health, particularly for marginalized religious groups. However, the extant empirical research on religion and population health remains emergent, particularly among adolescents (Kawachi, 2020; VanderWeele & Chen, 2019). Adolescence is an important developmental phase for examining these relationships given the salience of this period to identity formation, development of norms and values that can in turn influence behavior and lifestyle habits, as well as being a time of major biological growth and social role change (Hope et al., 2017).

Existing research on discrimination and adolescent health has predominantly focused on racial discrimination, and on mental health outcomes, with strong associations documented. The extent to which these associations exist for religious discrimination merits investigation. Moreover, although indicators of social emotional adjustment have been more widely studied within adolescent health research, there is burgeoning interest in sleep and sleep hygiene (behaviours that promote optimum development) as they are essential for daily functioning and health development (Priest et al., 2020; Yip, 2015; Yip et al., 2020). Sleep can act as either a risk or protective factor for a myriad of outcomes critical for adolescent wellbeing. For example, sleep disturbance is associated with poorer physical and psychological health outcomes. There is also growing evidence of associations with cardiovascular disease risk (Matthews & Pantesco, 2016; Priest et al., 2020).

Sleep is vital for health, well-being and cognitive functioning and therefore optimizing sleep behavior is becoming a growing public health priority. Moreover, poor sleep patterns (e.g. deficiency and sleep disorders) are common among children and young people (Keyes et al., 2015) and there is evidence doucmenting variations in sleep patterns among children by race and ethnicity (Hawkins & Takeuchi, 2016), with children from minoritized groups experiencing poorer sleep-associated health outcomes than their white peers (Guglielmo et al., 2018). A study of Australian children found that direct and vicarious experiences of racial discrimination were associated with sleep duration, sleep latency and sleep disruption (Priest et al., 2020). Among a diverse sample of American adolescents, discrimination was found to be associated with sleep disturbance, with ethnic and racial identity buffering the impact of discrimination on sleep (Yip et al., 2020). Furthermore, a recent systematic review (Cave et al., 2020) found that sleep duration was also a mediating variable along the pathway from racial discrimination to subsequent health outcomes. This demonstrates that more research is needed to understand the relationship between sleep and experiences of discrimination.

### 1.1 Overview of the study

This current study addresses these gaps in the literature by examining a) the prevalence of religious discrimination by religious affiliation and b) the relationship between religious discrimination and sleep quality and social emotional adjustment among a large, population representative ethnically diverse sample of school-aged adolescents in two of the largest states in Australia. Aboriginal and Torres Strait Islander people comprise 3.3 % of the total Australian population (Australian Bureau of Statistics, 2017). Moreover, Australia is undergoing major demographic changes contributing to the multi-ethnic composition of the population, with half (49 %) of respondents either born overseas or had a parent born outside Australia (ABS, 2017). About one-fifth (21 %) of the population speaks a language other than English at home. Religious diversity is growing in Australia; while many identify as Christian (52 %), a growing proportion of the population identify with “no religion” (30 %) and Islam and Buddhism are emerging as the second and third largest non-Christian religions (Bouma & Halafoff, 2017).

## 2 Methods

Data came from the Speak Out Against Racism (SOAR) 2017 Student Survey, a population representative, cross-sectional study of 4664 public school students in grades 5–9 (10–15 years of age) in two Australian states: New South Wales and Victoria. The self-administered survey covered a range of topics including: socio-demographic characteristics, health behaviors, perceptions of the school climate (e.g. perceived connectedness at school and with peers). In addition, this survey examined students’ experiences of discrimination based on their race/ethnicity, gender and religion as well as their attitudes towards race- and religion-based bullying among peers. Data were collected at 23 schools between May and August 2017 by trained researchers.

Details on the selection of schools is provided elsewhere (Priest et al., 2019). However, in brief, a list of government schools was obtained from each state's education department and schools were then stratified based on their proportion of: 1) Indigenous students and 2) students who spoke a language other than English. Schools with high proportions of Indigenous students were oversampled. Parental consent and student assent were required for participation and principals' approval from each school was obtained. Ethics committee approval was obtained from the Blinded for Review and from Blinded for Review.

Weights were created to adjust the sample to be representative of the government school student population in each state and to account for clustering at the school level. Weights were calculated for each responding student using the raking weighting method (Priest et al., 2019). implemented in statistical program R. A design weight was assigned for each respondent as the inverse of their chance of being selected to take part in the survey. The base weights were adjusted so that the relative frequencies of selected characteristics among respondents matched the population frequencies. The characteristics for which the adjustments were carried out were those involved in the selection process – Aboriginal and Torres Strait Islander tertile, Language Background Other than English, Occupation and Education Index category, and part of state (metropolitan, versus rest of state) (Priest et al., 2019).

### 2.1 Outcomes

#### 2.1.1 Social emotional adjustment

The Strengths and Difficulties Questionnaire (SDQ) is a brief questionnaire assessing the psychological adjustment of children and youth (Goodman, 2001). The youth SDQ (for children ages 11–17) consists of 25 items across five subscales. To provide a holistic profile of social-emotional adjustment, we included indicators of both optimal and adverse outcomes: 1) total difficulties, conduct and emotional scales indicative of externalizing and internalizing problems, respectively (Bayer et al., 2011) and 2) prosocial behavior indicative of child's positive social-emotional adjustment. The SDQ is not intended to be used as a diagnostic instrument; it indicates problematic emotions and behaviors across a range from normative to highly elevated (Stone et al., 2010) While cut-points have been developed for the SDQ these have not been validated for ethnic minority youth. Therefore, continuous scores are used for the current study following previous approaches in this study population and context (Priest et al., 2020).

#### 2.1.2 Sleep

*Sleep duration* was measured by students' response to a question about what time they fall asleep and wake up on a usual school day and on a non-school day. Sleep duration was calculated as the difference between reported sleep time and reported wake-up time, separately for school and non-school days. Analysis was restricted to sleep durations between 2.5 h and 20 h (Paine & Gander, 2016) which included 99.7 % of observations (n = 9 school day and n = 19 non-school day observations were excluded from analysis). Sleep difficulties were measured by examining *sleep latency* and *sleep disruption*. *Sleep latency* was measured using a single item “During the last four weeks, how long did it usually take for you to fall asleep”. A 3-category analytic variable was created: 0–30, 30–60, >60 min. *Sleep disruption* was measured using a single item ‘During the past four weeks, how often did you awaken during your sleep time and have trouble falling back to sleep again?’ A 3-category analytic variable was created: none/a little, some/a good bit, most/all. These items have previously been used with children and adolescents from diverse ethnic backgrounds (Paine & Gander, 2016).

### 2.2 Exposures

#### 2.2.1 Experiences of religious discrimination

Experiences of religious discrimination were measured using 10 items drawn from the Adolescent Discrimination Distress Index (ADDI)(Fisher et al., 2000) and two items used in a study on racism and racial attitudes among Australian school students (Priest et al., 2014) Items assessed discrimination by peers at school (4 items), by school personnel (3 items) and by others in the society (5 items). Each item was followed by the attribution (“because of …”) with “your religion” being one of three non-mutually exclusive options. Frequency of each experience was indicated from 0 = ‘this did not happen to me’, 1 = ‘once or twice’, 2 = ‘every few weeks’, 3 = ‘about once a week’, to 4 = ‘several times a week or more.’ Sub-scales were created for each source of discrimination (peer, school, societal) by calculating the mean score for relevant items as done previously (Fisher et al., 2000). Lastly, a total score was calculated by taking the average of responses to all 12 items.

#### 2.2.2 Covariates

Selection of covariates was based on theoretical and empirical studies and followed VanderWeele's definition of a confounder as a cause of exposure and/or outcome (VanderWeele, 2019). Other than Indigenous background, race or ethnicity is not commonly collected in national data collection efforts in Australia, including the Census. Ethnicity was measured using a self-reported variable with categories developed for the study. Students were provided several racial/ethnic categories to choose from (including checking off multiple) as well as an open-ended ‘other’ category that was later back coded. Following international approaches (Priest et al., 2019), a prioritization method was used to classify multiple responses to mutually exclusive categories based on level of stigmatization in Australia in the following order (Indigenous, Pacific Islander/Maori, Middle Eastern, African, Latinx, South Asian, East Asian, South East Asian, European and Anglo (White). Five per cent of students had missing ethnicity data due to ‘don't know’, unintelligible, or missing responses to this question. A ‘Missing’ ethnicity category was included in the analyses but estimates are not reported as meaningful interpretation was not possible. Due to very small numbers (n = 35) estimates for Latinx students are not reported. Gender was measured by response options: male, female and other. Country of birth was measured by dichotomizing whether students reported being born in Australia or in another (specified) country “Born outside Australia.” Religion was measured by the question: “What is your religion, even if you are not currently practicing?” Responses were combined into the following four categories: 1) No Religion 2) Christianity, 3) Islam, 4) Buddhism, 5) Hinduism and 6) Other. The “Other” category was comprised of a total of 18 religious groups and were aggregated due to the cell sizes within each group. Each student's grade was provided by the school. Index of Socioeconomic Advantage (ICSEA) is a continuous, composite variable comprised of parental occupation and education and school factors such as geographical location and proportion of Indigenous students (ACARA, 2013).

### 2.3 Analysis plan

Bivariate analyses (e.g. t-tests for continuous outcomes including SDQ and sleep duration and crosstabs for categorical outcomes including sleep latency and sleep disruption) were conducted to estimate the prevalence of key study variables by religious affiliation. Next, a series of regression models were fitted to examine the relationship between self-reported religious discrimination (peer, school, societal, total) and socioemotional (total difficulties, conduct, emotional, prosocial) and sleep outcomes (sleep duration, sleep latency, sleep disruption).

Linear regression models were fitted for socioemotional adjustment (continuous total difficulties, conduct, emotional, and prosocial scores) and sleep duration (continuous duration of sleep in minutes). Multinomial regression models were fitted and parametrized in terms of relative risk ratios for sleep latency (0–30 min vs 30–60 and 60 min) and sleep disruption (none/a little vs some/a good bit, and most/all trouble falling back to sleep). Unadjusted models examined the crude association between each racial discrimination exposure and each outcome. Next, models were further adjusted for gender, ethnicity, country of birth, year level and school socioeconomic background. As evidence suggests sleep problems may be on the causal pathway between discrimination and mental health, sleep was not included as a covariate in the socioemotional adjustment models.

All analyses were conducted in Stata version 15 using the ‘svy’ commands to account for the sampling weights and clustering at the school level.

## 3 Results

The socio-demographic characteristics (Table 1) demonstrate the diverse ethnic and religious composition of the sample. In parallel to the recent Census among adults (ABS, 2017), the largest proportion (approximately 56 %) of students identified as Anglo (40.0 %; 95 % CI 30.6, 49.1) or European (15.8 %; 95 % CI 12.3, 18.8). However, there was a diverse composition of students identifying with a non-Anglo/European ethnic group including: 4.6 % Indigenous (95 % CI 2.6, 8.0), 3.6 % Pacific/Maori (95 % CI 2.1, 6.3), 5.7 % Middle Eastern (95 % CI 3.6, 8.3), 4.1 % African (95 % CI 2.1, 4.7), 7.9 % East Asian (95 % CI (4.7, 12.3), 8.9 % South East Asian (95 % CI (3.0, 12.6) and 5.5 % South Asian (95 % CI 3.2, 9.0).

**Table 1:** Key study variables in the speak out against racism (SOAR) study, overall and by religious affiliation (4,664).

	% (95 % CI) or M(SD)	No Religion	Christian	Muslim	Buddhist	Hindu	Other
Religious Discrimination							
Peer **(%)**	21.3 (18.0, 25.2)	12.1 (9.1, 14.8)	21.1 (20.1, 28.9)	43.7 (30.7, 57.5)	27.0 (18.0, 38.5)	52.4 (31.3, 60.0)	37.8 (23.4, 54.8)
School **(%)**	10.5 (7.3, 13.7)	6.3 (3.1, 9.5)	12.3 (6.2, 14.7)	16.3 (9.1, 22.0)	15.8 (13.4, 19.7)	15.9 (11.2, 22.4)	31.7 (18.6, 41.3)
Societal **(%)**	16.3 (14.2, 19.4)	9.4 (7.2, 13.4)	16.6 (15.1, 19.7)	43.6 (32.1, 56.8)	27.3 (19.4, 38.5)	36.3 (28.5, 46.7)	36.9 (22.1, 55.5)
Total **(%)**	26.7 (22.8, 31.1)	15.4 (12.0, 19.5)	31.5 (28.6, 34.6)	56.5 (42.1, 69.9)	34.8 (26.7, 43.8)	57.3 (44.6, 69.2)	56.6 (32.5, 77.9)
**Sociodemographic Characteristics**							
Born overseas **(%)**	16.7 (13.2, 21.0)	8.4 (5.8, 11.9)	17.9 (12.7, 24.5)	46.0 (26.4, 66.3)	24.5 (12.3, 42.8)	47.0 (34.1, 60.3)	55.9 (29.9, 70.9)
Female **(%)**	51.0 (47.5, 54.5)	50.8 (47.5, 54.2)	54.4 (49.2, 59.5)	45.2 (39.3, 51.3)	49.3 (45.4, 53.1)	47.4 (37.3, 57.8)	39.1 (30.5, 48.5)
**Ethnicity**							
Indigenous **(%)**	4.6 (2.6, 8.0)	5.6 (2.8, 10.6)	4.8 (3.0, 7.5)	0.2 (0.0, 0.0)	1.4 (1.1, 1.9)	0.1 (0.00, 0.2)	3.1 (0.7, 8.1)
Pacific/Maori **(%)**	3.6 (2.1, 6.3)	1.9 (1.1, 3.3)	6.2 (3.7, 10.3)	2.5 (0.1, 10.6)	0.7 (0.1, 1.1)	1.9 (0.1, 6.2)	17.9 (6.0, 33.4)
Anglo **(%)**	40.0 (30.6, 49.1)	56.2 (49.7, 62.6)	35.2 (27.3, 43.9)	0.8 (0.1, 5.0)	2.1 (0.4, 9.5)	8.5 (2.5, 12.3)	14.7 (5.8, 21.2)
African **(%)**	4.1 (2.1, 4.7)	1.8 (0.0, 3.3)	5.1 (3.1, 8.4)	7.2 (3.2, 15.4)	0.5 (0.2, 8.1)	3.1 (0.00, 12.1)	2.6 (0.08, 7.5)
East Asian **(%)**	7.9 (4.7, 12.3)	6.4 (3.7, 10.7)	7.6 (4.0, 14.0)	0.5 (0.0, 3.1)	25.0 (15.7, 37.2)	0.1 (0.0, 1.0)	1.1 (0.3, 4.0)
	**% (95 % CI) or M(SD)**	**No Religion**	**Christian**	**Muslim**	**Buddhist**	**Hindu**	**Other**
European **(%)**	15.8 (12.3, 18.8)	16.0 (12.6, 20.2)	20.9 (16.7, 25.8)	1.2 (0.0, 4.2)	0.8 (0.1, 3.9)	0.4 (0.0, 2.9)	0.5 (0.0, 4.6)
Middle Eastern **(%)**	5.7 (3.6, 8.3)	0.6 (0.0, 1.4)	4.7 (1.1, 11.4)	59.4 (49.7, 68.5)	0.2 (0.0, 1.8)	0	15.1 (6.7, 23.5)
South Asian **(%)**	5.5 (3.2, 9.0)	0.9 (0.5, 1.7)	2.6 (1.5, 4.5)	20.3 (11.1, 34.3)	5.5 (1.7, 11.3)	84.4 (81.2, 87.0)	28.7 (12.9, 41.2)
South East Asian **(%)**	8.9 (3.0, 12.6)	3.0 (1.1, 8.3)	8.4 (5.4, 12.8)	4.02 (1.0, 14.8)	62.6 (42.7, 79.0)	0.7 (0.1, 3.8)	4.2 (1.0, 13.1)
**Health Outcomes**							
**Socioemotional adjustment**							
Total difficulties M(SD)	11.8 (6.3)	11.8 (6.5)	11.6 (6.1)	10.9 (5.9)	12.8 (4.7)	9.4 (4.7)	12.3 (8.9)
Emotional symptoms M(SD)	3.4 (2.3)	3.7 (2.4)	3.4 (2.2)	3.1 (2.2)	3.9 (1.9)	2.7 (2.3)	3.2 (2.9)
Conduct problems M(SD)	2.0 (1.8)	1.9 (1.9)	1.9 (1.8)	2.0 (1.6)	2.4 (1.8)	1.4 (1.1)	2.5 (2.7)
Prosocial behavior M(SD)	7.7 (1.8)	7.69 (1.8)	7.9 (1.7)	7.8 (1.9)	6.9 (1.9)	7.7 (1.6)	7.48 (2.0)
**Sleep latency**							
0–30 min **(%)** (base outcome)	63.2 (60.9, 65.5)	61.1 (58.2.64.3)	62.1 (56.2, 68.4)	75.1 (67.2, 82.4)	69.1 (59.1, 77.3)	80.4 (68.7, 88.4)	67.4 (59.1, 74.7)
>30–60 min **(%)**	22.2 (19.8, 24.8)	23.5 (22.8, 25.3)	22.8 (18., 29.7)	18.5 (14.3, 25.4)	16.4 (12.2, 22.4)	15.1 (9.5, 23.1)	24.4 (17.3, 33.2)
>60 min **(%)**	14.4 (13.0, 16.0)	15.3 (0.13, 0.18)	14.9 (13.0, 18.6)	6.2 (3.0, 12.1)	14.4 (10.6, 9.8)	4.5 (10.1, 17.3)	8.1 (3.8, 16.6)

	**% (95 % CI) or M(SD)**	**No Religion**	**Christian**	**Muslim**	**Buddhist**	**Hindu**	**Other**
**Sleep disruption**							
None/A little **(%)** (base outcome)	54.1 (52.1, 57.6)	54.4 (50.3, 58.4)	52.5 (47.4, 57.5)	51.4 (40.9, 61.8)	58.0 (44.3, 70.5)	79.6 (46.7, 94.5)	51.0 (38.9, 63.0)
Some/A good bit of the time **(%)**	26.1 (25.0, 27.2)	25.6 (23.5, 27.8)	27.3 (23.8, 31.1)	29.2 (21.4, 38.4)	23.5 (18.9, 28.9)	16.6 (4.4, 26.4)	30.2 (19.7, 43.2)
Most/All of the time **(%)**	19.0 (17.2, 23.3)	19.8 (16.5, 23.6)	20.1 (17.3, 23.1)	19.2 (16.1, 22.9)	18.3 (8.5, 35.1)	3.6 (1.1, 8.6)	18.7 (9.1, 23.6)
**Sleep duration**							
School day (minutes)	549.8 (85.3)	558.5 (86.2)	549.3 (79.8)	536.0 (104.3)	495.5 (76.8)	568.3 (57.5)	534.7 (108.7)
Non-school day (minutes)	583.1 (113.9)	582.8 (118.9)	586.1 (109.8)	576.1 (110.8)	575.51 (90.6)	601.8 (97.0)	557.06 (185.6)

Note: Percentages may not sum to 100 due to rounding.

When asked to report religious affiliation students largely reported “No Religion” (45.4 %; 95 % CI 33.2, 58.3) followed by Christianity (35.3 %; 95 % CI 28.7, 42.5), 6.2 % (95 % CI 1.4, 22.4) Buddhist, 4.9 % (95 % CI 2.9, 8.1) Muslim, 2.4 % (95 % CI 1.0, 5.6) Hindu and 1.7 % (95 % CI 1.0, 2.8) “Other.” Approximately 17 % of students (95 % CI 13.2, 21.0) were born overseas. It is noteworthy to highlight that a large proportion of students from non-Christian religious groups were foreign-born. Specifically, 46 % (95 % CI 26.4, 66.3) of Muslim, 24.5 % (95 % CI 12.3, 42.8) of Buddhist, 47 % (95 % CI 34.1, 60.3) of Hindu and 55.9 % (95 % CI 29.9, 70.9) of students identifying with an “Other” religion were born outside of Australia.

Overall, over a quarter (26.7 %; 95 % CI 22.8, 31.1) of students reported experiences of direct total religious discrimination, including direct experiences from peers (21.3 % 95 % CI 18.0, 25.2), school (10.5 %; 95 % CI 7.3, 3.7) and societal (16.3 % 95 % CI 14.2, 19.4) sources. Across all sources of religious discrimination, far greater proportions of students who identified as Muslim, Buddhist, Hindu or Other, reported religious discrimination experiences than those students who identified with the dominant “No Religion” group (see Table 1).

There was strong evidence that experiences of religious discrimination across all four sources was related to socioemotional adjustment, after adjusting for socio-demographic characteristics (Table 2). In other words, each increase in frequency of total direct religious discrimination was associated with an increase of 3.7 (95 % CI 2.8, 4.5) in total difficulty scores. This effect was also detected when examining emotional symptoms, such that each increase in frequency of reporting experiencing total direct religious discrimination was associated with an increase of 0.9 in the score measuring (adverse) emotional symptoms (b = 0.9, 95 % CI 0.6, 1.2), and also with an increase in the score measuring conduct problems (b = 1.0, 95 % CI 0.6, 1.4). However, an increase in reporting religious discriminatory experience was associated with a decrease of 0.3 (95 % CI -0.5, −0.1) in the score assessing prosocial behavior.

**Table 2:** Estimates from linear regression models showing associations between self-reported religious discrimination and socioemotional adjustment in the Speak Out Against Racism (SOAR) Study (N = 4480).

	Total difficulties		Emotional symptoms		Conduct problems		Prosocial behavior	
Religious Discrimination	Unadjusted b (95 % CI)	Adjusted^#^ b (95 % CI)	Unadjusted b (95 % CI)	Adjusted^#^ b (95 % CI)	Unadjusted b (95 % CI)	Adjusted^#^ b (95 % CI)	Unadjusted b (95 % CI)	Adjusted^#^ b (95 % CI)
Peer	3.1 (2.6, 3.5)	3.1 (2.5, 3.7)	0.7 (0.5, 0.9)	0.7 (0.5, 1.0)	0.9 (0.6, 1.1)	0.8 (0.5, 1.0)	−0.2 (−0.3, −0.1)	−0.2 (−0.3, −0.1)
School	3.0 (2.3, 3.7)	2.8 (1.9, 3.7)	0.6 (0.3, 0.8)	0.6 (0.3, 0.9)	1.0 (0.6, 1.4)	0.9 (0.5, 1.2)	−0.4 (−0.6, −0.1)	−0.3 (−0.7, −0.0)
Societal	3.4 (2.8, 3.9)	3.2 (2.6, 3.9)	0.8 (0.6, 1.1)	0.9 (0.6, 1.1)	1.0 (0.6, 1.3)	0.9 (0.5, 1.2)	−0.3 (−0.5, −0.1)	−0.2 (−0.5, −0.0)
Total direct	3.8 (3.1, 4.4)	3.7 (2.8, 4.5)	0.8 (0.6, 1.1)	0.9 (0.6, 1.2)	1.1 (0.7, 1.5)	1.0 (0.6, 1.4)	−0.3 (−0.5, −0.1)	−0.3 (−0.5, −0.1)

^#^Adjusted for ethnicity, gender, religion, school year, country of birth, ICSEA.

Strong evidence was also found for an effect of direct religious discrimination across almost all sleep outcomes. An increase in the frequency of reporting experiencing religious discrimination, across all four sources of discrimination, was associated with shorter sleep duration on school days (Table 3). For example, after adjusting for socio-demographic characteristics, each 1-point increase in total direct religious discrimination was associated with approximately 20 (b = 19.8, 95 % CI -36.0, −3.6) fewer minutes of sleep on a school day. However, there were no associations between any of the measures of religious discrimination on sleep duration on non-school days (Table 3). Similarly, after adjusting for socio-demographic characteristics, each 1-point increase in total direct religious discrimination was associated with 1.7 times the risk of (95 % CI 1.3, 2.2) sleep latency greater than 60 min in comparison to sleep latency spanning 0–30 min (Table 4). Lastly, after controlling for socio-demographic characteristics, a 1-point increase in total direct experiences of religious discrimination was associated with 2.4 the risk (95 % CI 1.3, 4.4) of reporting sleep disruption most or all of the time in comparison to none of the time or a little of the time (Table 4).

**Table 3:** Estimates from linear regression models showing associations between self-reported religious discrimination and sleep duration in the Speak Out Against Racism (SOAR) Study (N = 3997).

	Sleep duration (minutes)			
	School day		Non-school day	
**Religious Discrimination**	Unadjusted b (95 % CI)	Adjusted^#^ b (95 % CI)^#^	Unadjusted b (95 % CI)	Adjusted^#^ b (95 % CI)^#^
Peer	−15.1 (−25.8, −4.4)	**−15.1** (−27.4, −2.8)	−4.3 (−12.3, 3.7)	−2.0 (−11.9, 7.8)
School	−16.5 (−30.8, −2.1)	**−13.9** (−27.1, −0.7)	−4.1 (−10.7, 2.5)	0.6 (−6.2, 7.4)
Societal	−20.2 (−35.1, −5.3)	−**19.0** (−34.2, −3.7)	−2.7 (−12.4, 6.8)	−0.0 (−11.5, 11.5)
Total direct	−20.8 (−36.0, −5.6)	**−19.8** (−36.0, −3.6)	−3.2 (−12.4, 5.8)	0.3 (−10.9, 11.5)

^#^Adjusted for ethnicity, gender, religion, school year, country of birth, ICSEA.

**Table 4:** Multinomial logistic regression showing associations between self-reported religious discrimination and sleep difficulties in the Speak Out Against Racism (SOAR) Study (N = 4118).

	Sleep latency				Sleep disruption			
	>30–60 min vs 0–30 min		>60 min vs 0–30 min		Some/A good bit vs None/A little		Most/All of the time vs None/A little	
**Religious Discrimination**	Unadjusted RR (95 % CI)	Adjusted^#^ RR (95 % CI)	Unadjusted RR (95 % CI)	Adjusted^#^ RR (95 % CI)	Unadjusted RR (95 % CI)	Adjusted^#^ RR (95 % CI)	Unadjusted RR (95 % CI)	Adjusted^#^ RR (95 % CI)
Peer	1.1 (0.9, 1.2)	**1.2** (1.0, 1.3)	1.4 (1.2, 1.7)	**1.6** (1.3, 1.9)	1.5 (1.1, 2.0)	**1.7** (1.1, 2.5)	1.7 (1.4, 2.1)	**1.9** (1.3, 2.6)
School	1.0 (0.8, 1.3)	1.1 (0.9, 1.4)	1.4 (1.0, 2.0)	**1.4** (1.1, 2.0)	1.4 (0.9, 2.1)	1.5 (0.9, 2.5)	1.9 (1.2, 2.9)	**1.9** (1.1, 3.5)
Society	1.0 (0.8, 1.3)	**1.1** (0.9, 1.4)	1.6 (1.2, 1.9)	**1.6** (1.3, 2.1)	1.7 (1.1, 2.5)	**1.8** (1.1, 3.0)	2.3 (1.4, 3.6)	**2.3** (1.3, 4.0)
Total direct	1.0 (0.9, 1.2)	**1.2** (1.0, 1.4)	1.6 (1.3, 2.0)	**1.7** (1.3, 2.2)	1.8 (1.2, 2.7)	**1.9** (1.1, 3.4)	2.3 (1.5, 3.5)	**2.4** (1.3, 4.4)

^#^ Adjusted for ethnicity, gender, religion, country of birth and ICSEA.

## 4 Discussion

The goal of this study was to build on the understudied relationship between religious discrimination and indicators of adolescent health and wellbeing including social emotional adjustment and sleep behaviors. Within the study's sample, over one-quarter (27 %) of students reported experiencing total religious discrimination. Students from minoritized religious groups (e.g. Islam, Buddhism) reported higher levels of discrimination across all sources than their peers who identified as either “no religion” or Christian.

The results suggest that religious discrimination had a deleterious impact on the health and wellbeing of adolescents, irrespective of whether it was from peers, elsewhere in the school, or from wider societal interactions. An increase in experiences of religious discrimination (across all four sources) was associated with an increase in total difficulties, emotional symptoms, and conduct problems but negatively associated with prosocial behavior. Our findings are consistent with, but also add to, the existing body of literature on the adverse impact of racial discrimination on indicators of social-emotional wellbeing outcomes among adolescents (Cogburn et al., 2011; Priest et al., 2020). For example, in a recent study of adolescents in the U.S., religious discrimination had a negative impact on the psychological and mental health of adolescents identifying with a marginalized religious and racial and ethnic minority group (Balkaya et al., 2019).

To our knowledge, this is the first study to examine the relationship between religious discrimination and sleep behavior among adolescents. Experiences of religious discrimination were associated with fewer minutes of sleep on school days only. In addition, an increase in reported experiences of religious discrimination was associated with higher risk of sleep latency as well as higher risk of frequent sleep disruptions. Our findings align with several studies documenting negative associations between racial discrimination and sleep quality (Majeno et al., 2018; Zeiders, 2017). For example, among a sample of ethnically diverse adolescents experiences of discrimination, whether attributed to ethnicity or other characteristics, was found to be negatively associated with perceived sleep quality, particularly sleep disturbances (Majeno et al., 2018). Thus, other forms of discrimination, not just discrimination attributed to one's ethnicity, are detrimental to sleep quality.

There is growing interest in examining how the school social environment can have implications for students' wellbeing and health (Carta et al., 2015; Eccles & Roeser, 2012; Powell et al., 2018). And in the current study, an interesting relationship emerged between religious discrimination and fewer minutes slept held true on school days, but not on non-school days. A prior study among Mexican-origin young adults in the United States described that fluctuations in sleep may be impacted by experiences of discrimination across different settings (e.g. school, community) that can activate feelings of threat or arousal thereby impeding sleep on some nights and cause need for catch up sleep on other nights (Zeiders, 2017). Moreover, the author hypothesized that perceived discrimination experienced at school elicits “rumination processes and or vigilance against threat” which can disrupt sleep patterns and quality (Zeiders, 2017). Thus, a school setting that is hostile, or discriminatory, to students from certain religious backgrounds could be one contributing factor to the patterns in our sample such that the students who experience more religious discrimination at school have poorer sleep quality on school days than on non-school days. Moreover, it could be that the non-school days provide these students with more time in other contexts (e.g. home or community) in which their religious identities are affirmed and/or not threatened which then can promote optimal sleep practices. This aligns with a recent study in the U.S. (Montoro et al., 2021) that found a negative impact of experiences of racial discrimination at school on student's sense of belonging and their academic performance. Thus, the current study adds to the growing body of literature emphasizing the critical role the school environment can be for increasing exposure to risk factors, such as discrimination, that have implications for childrens' development and health (Eccles & Roeser, 2012; Huang et al., 2013) considering the large proportion of time youth spend at school (Carta et al., 2015; Powell et al., 2018). Specifically, the findings provide evidence on how religious discrimination within the school context is associated with shorter sleep duration, an understudied outcome among child health disparities research.

### 4.1 Implications for future research and practice

Overall, our findings support the growing evidence that stressors within the social environment, including religious discrimination, impact adolescent health and development and that ongoing research is needed that takes a holistic approach, examining multiple outcomes, towards investigating these relationships. The results, together with the global rise in religion-based hate crimes, underscore the need for more research conceptualizing and addressing religious discrimination as a form of stress that poses risks to population health and health disparities, and especially so among religious minorities in Western contexts. For example, a study among British Muslim school students argued that racism experienced by Muslim students often included accounts of religious discrimination, something which is not often considered in discussions and scholarship about racism (Gilbert, 2004) and health. Additionally, future research is needed to help delineate the pathways in the observed association between discriminatory experiences within the school context and sleep behavior. Future research can also guide the development of anti-discriminatory programs and policies within the school setting that create learning environments that are supportive and inclusive to students of all religious and racial/ethnic backgrounds.

### 4.2 Limitations

There are limitations to the study that should be considered when interpreting findings. First, the data are all based on students’ self-report and are therefore subject to biases including social desirability and recall. More objective measures of sleep and social emotional adjustment would have bolstered the data as would have student-level data measuring their socioeconomic background. Also, there are additional, unmeasured, factors that this study did not include (e.g. religiosity) that could influence the main relationship of interest. Third, the study is cross-sectional and therefore is limited in testing potential pathways between discrimination, sleep and social-emotional outcomes and determining the directionality of relationships.

## 5 Conclusion

Albeit understudied, religious discrimination is a highly prevalent form of everyday social disadvantage that has implications for health and should be more strongly considered in population health research. This study is one of the first to document a relationship between religious discrimination and two indicators of health and wellbeing among adolescents: social emotional adjustment and sleep. The results clearly signify the urgent need for more research, policies and programs to curtail the impact religious based discrimination can have on adolescent health and development.

## Author statement

Sharif led the conceptualization, supervision and writing of the manuscript. Priest led the formal analyses. All authors contributed to the writing, review and editing of the manuscript.

## Ethical statement

None of the authors have any conflicts of interest to disclose. All authors were involved in the various stages of manuscript preparation and they have all approved the submitted manuscript.

Speak out Against Racism (SOAR) was conducted in partnership with the New South Wales and Victorian education departments and the Australian Human Rights commission. The study sponsors had no role in study design; the collection, analysis, and interpretation of data; the writing of the report; or the decision to submit the manuscript for publication. The authors would like to thank all schools and students participating in SOAR. We thank Tania King for her work during the early stages of SOAR and research staff (Rebecca Moorhead, Sharon Moorhead, Brandi Fox, Meiliasari Meiliasari and Emma Whatman (Victoria); and Oishee Alam, Alexia Derbas, Katie Blair, Rosalie Atie and Zarlasht Sarwari (New South Wales)) who were involved in data collection. We acknowledge the support of the Social Research Centre with data collection.
